# *In Vitro* Cultivation of ‘Unculturable’ Oral Bacteria, Facilitated by Community Culture and Media Supplementation with Siderophores

**DOI:** 10.1371/journal.pone.0146926

**Published:** 2016-01-14

**Authors:** Sonia R. Vartoukian, Aleksandra Adamowska, Megan Lawlor, Rebecca Moazzez, Floyd E. Dewhirst, William G. Wade

**Affiliations:** 1 Barts and The London School of Medicine and Dentistry, Queen Mary University of London, London, United Kingdom; 2 King’s College London Dental Institute, London, United Kingdom; 3 The Forsyth Institute, Cambridge, United States of America; 4 Harvard School of Dental Medicine, Boston, United States of America; LSU Health Sciences Center School of Dentistry, UNITED STATES

## Abstract

Over a third of oral bacteria are as-yet-uncultivated in-vitro. Siderophores have been previously shown to enable in-vitro growth of previously uncultivated bacteria. The objective of this study was to cultivate novel oral bacteria in siderophore-supplemented culture media. Various compounds with siderophore activity, including pyoverdines-Fe-complex, desferricoprogen and salicylic acid, were found to stimulate the growth of difficult-to-culture strains *Prevotella* sp. HOT-376 and *Fretibacterium fastidiosum*. Furthermore, pyrosequencing analysis demonstrated increased proportions of the as-yet-uncultivated phylotypes *Dialister* sp. HOT-119 and *Megasphaera* sp. HOT-123 on mixed culture plates supplemented with siderophores. Therefore a culture model was developed, which incorporated 15 μg siderophore (pyoverdines-Fe-complex or desferricoprogen) or 150 μl neat subgingival-plaque suspension into a central well on agar plates that were inoculated with heavily-diluted subgingival-plaque samples from subjects with periodontitis. Colonies showing satellitism were passaged onto fresh plates in co-culture with selected helper strains. Five novel strains, representatives of three previously-uncultivated taxa (*Anaerolineae* bacterium HOT-439, the first oral taxon from the *Chloroflexi* phylum to have been cultivated; *Bacteroidetes* bacterium HOT-365; and *Peptostreptococcaceae* bacterium HOT-091) were successfully isolated. All novel isolates required helper strains for growth, implying dependence on a biofilm lifestyle. Their characterisation will further our understanding of the human oral microbiome.

## Introduction

The human oral microbiome is composed of a diverse community of bacteria, fungi, protozoa, viruses and archaea [1]. When the symbiotic relationship between the oral microbiota and host breaks down, a dysbiotic microbial community is established, a change associated with two of the commonest bacterial diseases of man: periodontitis (gum disease) and dental caries (tooth decay) [2]. Recent evidence indicates that oral bacteria also have a significant impact on systemic health and disease. For example, it has been shown that oral bacteria are involved in reduction of dietary nitrate and consequent release of nitric oxide systemically, with a resultant blood-pressure lowering effect [3].

The advent of next generation sequencing has led to a better appreciation of the true diversity of the human oral bacteriome, and release 13 of the Human Oral Microbiome Database (HOMD) (www.homd.org) lists approximately 700 taxa at species level. However, over a third of these, nearly 250, are as yet uncultivated and primarily known only by 16S rRNA gene sequence data [4, 5]. Members of the candidate bacterial Divisions SR1 and GN02 have been frequently detected in molecular 16S rDNA analyses of human (oral) and other samples, but none have ever been cultivated *in vitro* [6]. Based on molecular analyses, representatives of the TM7 phylum have been found in high prevalence in the oral cavity, and yet, until the recent cultivation of strain TM7x [7], there were no cultivated members. Although the phylum *Chloroflexi* includes several cultivable species, no taxon found in the human oral cavity has been successfully cultivated [6].

An exploration of the possible reasons for bacteria being uncultivable in the laboratory has been reviewed previously [8, 9]. Arguably, the primary reason for the resistance of bacteria to *in vitro* cultivation (in isolation) is a dependence on other bacteria and on chemical signals within mixed communities. This may be particularly relevant to bacteria inhabiting biofilm-type communities, such as dental plaque, where metabolic cooperation and intercellular signaling networks are widespread [10]. Furthermore, the sharing of metabolites, such as iron-chelating siderophores, within bacterial communities is prevalent particularly in bacteria-host environments [11, 12], and it has been suggested that the ability to produce such siderophores may have been lost in certain ‘unculturable’ bacteria [13].

In light of the above, efforts to cultivate ‘unculturable’ bacteria have sometimes focused on using either a ‘simulated natural environment’ [14, 15], adding chemical factors that are thought to be required (but not produced) by difficult-to-grow bacteria to culture media [16, 17], or using co-culture with other bacteria from the same community to facilitate growth of dependent strains [18, 19].

Despite a wealth of information having been gained in recent years through metagenomic, metaproteomic, metatranscriptomic and other ‘meta-omic’ analyses of microbial communities [20], it is only through the isolation and culture of individual species that phenotypic and genotypic characteristics can be comprehensively characterised, and virulence potential systematically investigated. On the basis that as-yet-uncultivated bacteria are equally likely to play a role in disease processes as are previously-cultivated bacteria, it is clear that the quest to cultivate them is a most important one.

The aims of this study were: (a) to investigate various siderophore-like agents for a growth-stimulatory effect on ‘difficult-to-culture’ oral bacteria; and (b) to develop an in-vitro culture model for the cultivation and isolation of previously-uncultivated oral bacteria, using various means, including community culture and provision of siderophores as potential growth supplements. The growth of difficult-to-culture oral bacteria *Prevotella* sp. HOT-376 and *Fretibacterium fastidiosum* was found to be stimulated by various siderophores. Five novel, previously-uncultivated bacterial strains were successfully isolated using our in-vitro culture model.

## Results

### Growth stimulation of ‘difficult-to-culture’ oral bacterial strains by siderophores

The ability of six siderophore-like/iron-chelating agents to stimulate the growth of *Prevotella* sp. HOT-376 (KCL-E7_34) and *Fretibacterium fastidiosum*, both of which are unable to grow in pure culture and depend for growth on helper strains such as *Fusobacterium nucleatum*, was investigated using lawn cultures, with test agents placed in a central well in each plate.

**Fig 1:**
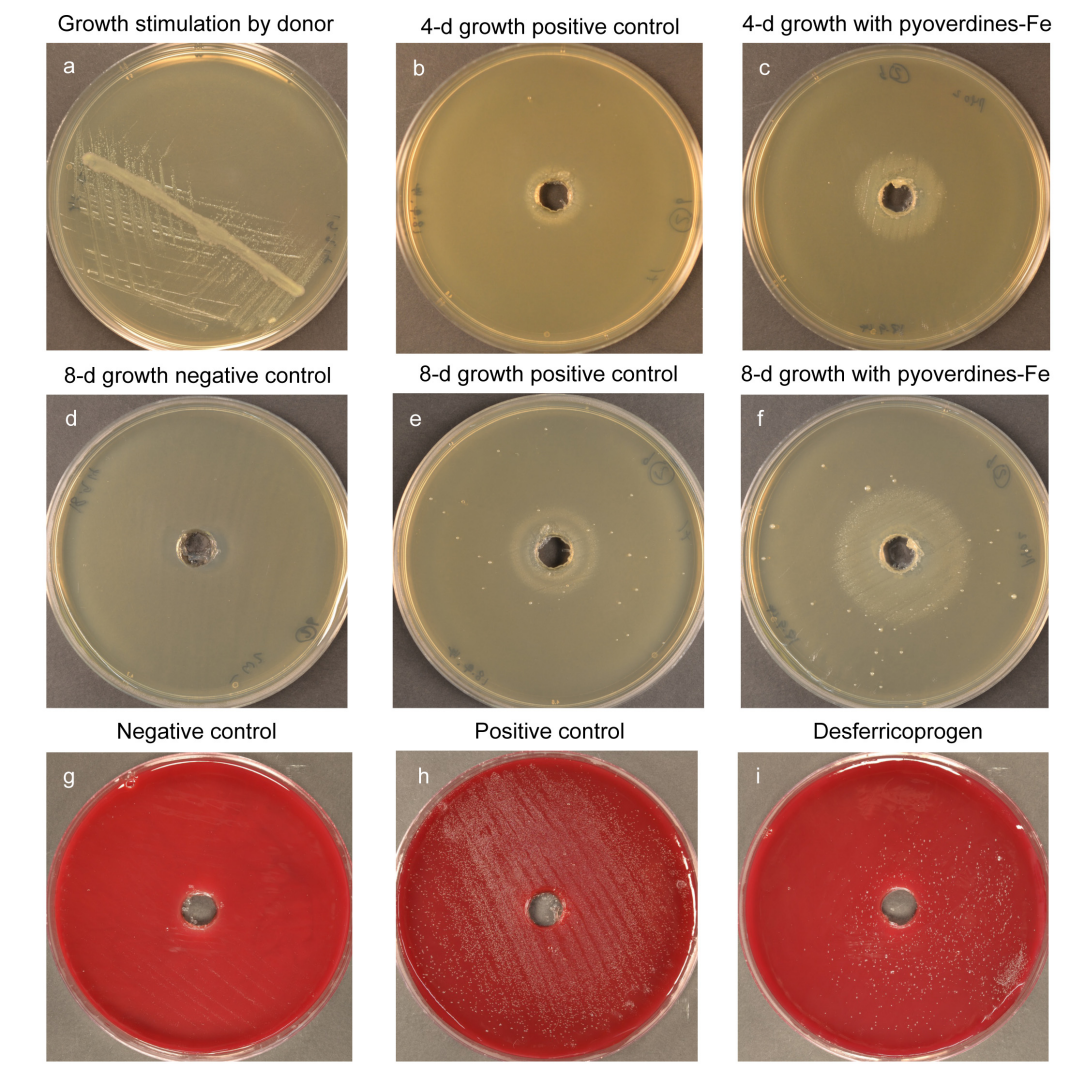
Growth stimulation of ‘difficult-to-culture’ oral bacterial strains by siderophores. (a-f) Growth of *Prevotella* sp. HOT-376: (a) *Fusobacterium nucleatum* cross-streak, (b-f) 4- & 8-d plates with pyoverdines-Fe, *F*. *nucleatum* culture supernatant (positive control) or water (negative control); (g-i) *Fretibacterium fastidiosum*: 28-day spread plates supplemented with desferricoprogen, positive or negative control solutions (as described above).

Growth of *Prevotella* sp. HOT-376 was stimulated by *F*. *nucleatum* culture filtrate (positive control) with a mean growth zone radius of 13.4 mm after eight days (Fig 1E), whilst pyoverdines-Fe at 0.1 mg/ml stimulated growth more strongly, with a mean zone of stimulation of 22.7 mm (Fig 1F). 2,3-dihydroxybenzoic acid exhibited no growth stimulation. The other test agents enhanced the growth of *Prevotella* sp. HOT-376 compared to corresponding negative controls (agent diluents), but to a lesser degree than the positive control (Table 1).

**Table 1:** Growth stimulation of *Prevotella* sp. HOT-376 and *Fretibacterium fastidiosum* by test agents relative to positive (++++) and negative (-) controls.

Test agent	*Prevotella* sp. HOT-376	*Fretibacterium fastidiosum*
Desferricoprogen	+	++
Pyoverdines-Fe	+++++	-
Ferrichrome-Fe-free	+	+
2,3-Dihydroxybenzoic acid	-	+/-
Salicylic acid	+	++
Ferric citrate	++	-

The positive control stimulated growth of *F*. *fastidiosum* resulting in a moderately dense growth over the entire inoculated surface after 28 d (Fig 1H). Of the agents tested, desferricoprogen and salicylic acid consistently showed the greatest growth enhancement of *F*. *fastidiosum* relative to the negative control (Table 1), giving rise to a sparse growth of colonies up to the periphery of the agar (Fig 1I).

### Development of an in-vitro culture model for the cultivation and isolation of previously-uncultivated oral bacteria

Pyoverdines-Fe, desferricoprogen or an undiluted suspension of plaque were used to stimulate growth of previously-uncultivated oral bacteria in heavily-diluted samples of subgingival plaque (from below the gum line), inoculated onto agar culture plates.

**Fig 2:**
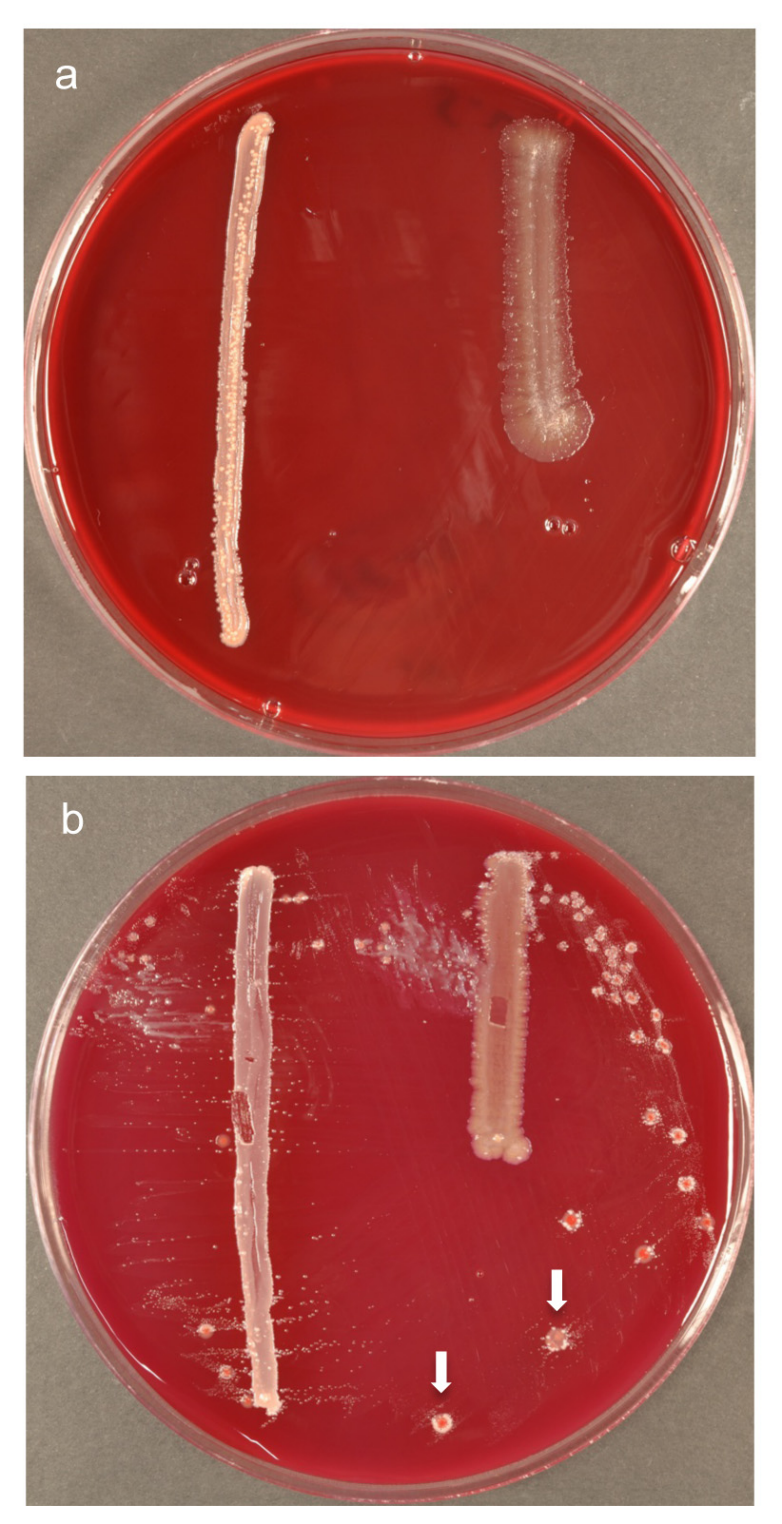
Secondary cultures of colonies of interest. (a) Growth of an unknown strain limited to the surface of a *Propionibacterium acnes* cross-streak (on left); (b) Secondary culture of *Bacteroidaceae* bacterium HOT-272 showing satellitism beside the *P*. *acnes* cross-streak (on left) and around red colonies of *Actinomyces odontolyticus* (indicated with arrows).

In the first round of experimentation using plaque cultures of samples harvested from deep periodontal pockets of two subjects with chronic periodontitis (SP9 and SP10), 31 colonies of interest (microcolonies/colonies showing satellitism) were identified on primary plates (of 10^6^-diluted samples) at eight or more days of growth; none of these colonies of interest originated from ‘negative control’ plates that were supplemented only with water. Ensuring that primary plates were not outside the anaerobic cabinet for more than 30 minutes at a time, colonies of interest were passaged onto plates that were: pre-reduced in an anaerobic atmosphere at room temperature for two hours, cross-streaked with potential helper strains (*Propionibacterium acnes*, *F*. *nucleatum*, or colonies found in close proximity on primary plates), and where appropriate, supplemented with either pyoverdines-Fe or desferricoprogen. No growth was seen (as viewed under a dissecting microscope) on plates derived from 12 (39%) of the original colonies of interest. A further eight (26%) were found only to grow on the surface of *P*. *acnes* cross-streaks (Fig 2A). Some of the remaining secondary cultures resulted in a single isolated colony and others showed evidence of satellitism (Fig 2B). Where necessary for purification, cultures were further sub-cultured, the 16S rRNA gene sequence of isolates was analysed, and purity of cultures confirmed by sequencing 20 clones per library prepared from a single colony. As a result, two strains (SP9_5 and SP10_2) of the previously-uncultivated *Chloroflexi* phylum oral taxon *Anaerolineae* bacterium HOT-439 were successfully isolated in culture. Additionally, two strains (SP9_3 and SP10_10) of the previously-cultivated but unnamed taxon *Bacteroidaceae* bacterium HOT-272 were isolated.

Alternative means of incorporating helper strains into the culture system were evaluated. The growth of dependent strains (*Chloroflexi* HOT-439 and *Bacteroidaceae* HOT-272) on the surface of sterile membranes overlying helper lawns (*F*. *nucleatum* and *P*. *acnes* respectively) was assessed, using a selection of different membrane materials (nitrocellulose or cellulose acetate), membrane pore sizes (0.45 μm or 0.22 μm) and lag-times between preparation of helper lawns and inoculation of membranes with recipients (0, 4 or 48 hours). 0.45 μm-pore, cellulose acetate membranes (Sartorius, 1110650ACN) placed over fresh *F*. *nucleatum* or 48-hour *P*. *acnes* lawns were found to be optimal for growth, resulting in significantly greater numbers of colonies at 21 days of incubation than with any of the other conditions tested. This regime was followed for all subsequent ‘membrane’ cultures.

In a second round of experimentation with plaque samples from a further two subjects with chronic periodontitis (SP18 and SP19), the original protocol for passage was modified to include membrane cultures (as above), in addition to cross-streaked plates. A total of 46 colonies of interest were identified on the ‘10^6^-dilution’ primary plates supplemented with plaque, pyoverdines-Fe or desferricoprogen. There was no visible growth on secondary plates derived from 18 (39%) of these, but this time only one colony (2%) showed growth limited to the surface of the *P*. *acnes* cross-streak. Of the remaining target colonies, some showed evidence of weak or dependent growth on subculture, while others were found to grow independently. Previously-uncultivated taxa isolated during the second experiment included: *Chloroflexi* taxon *Anaerolineae bacterium* HOT-439 (strain SP19_9), *Bacteroidetes* bacterium HOT-365 (strain SP18_29), and *Peptostreptococcaceae* bacterium HOT-091 (strain SP19_5). Additionally, one strain (SP18_6) of the previously-cultivated but unnamed taxon *Capnocytophaga* sp. HOT-336 was isolated.

Two of the three isolated *Chloroflexi* taxon *Anaerolineae* bacterium HOT-439 strains (SP9_5 and SP10_2) were originally detected on plaque-supplemented plates, and strain SP19_9, on a desferrricoprogen plate; the *Bacteroidetes* bacterium HOT-365 strain was found on a pyoverdines-Fe plate; and *Peptostreptococcaceae* bacterium HOT-091, on a plaque-supplemented plate.

**Fig 3:**
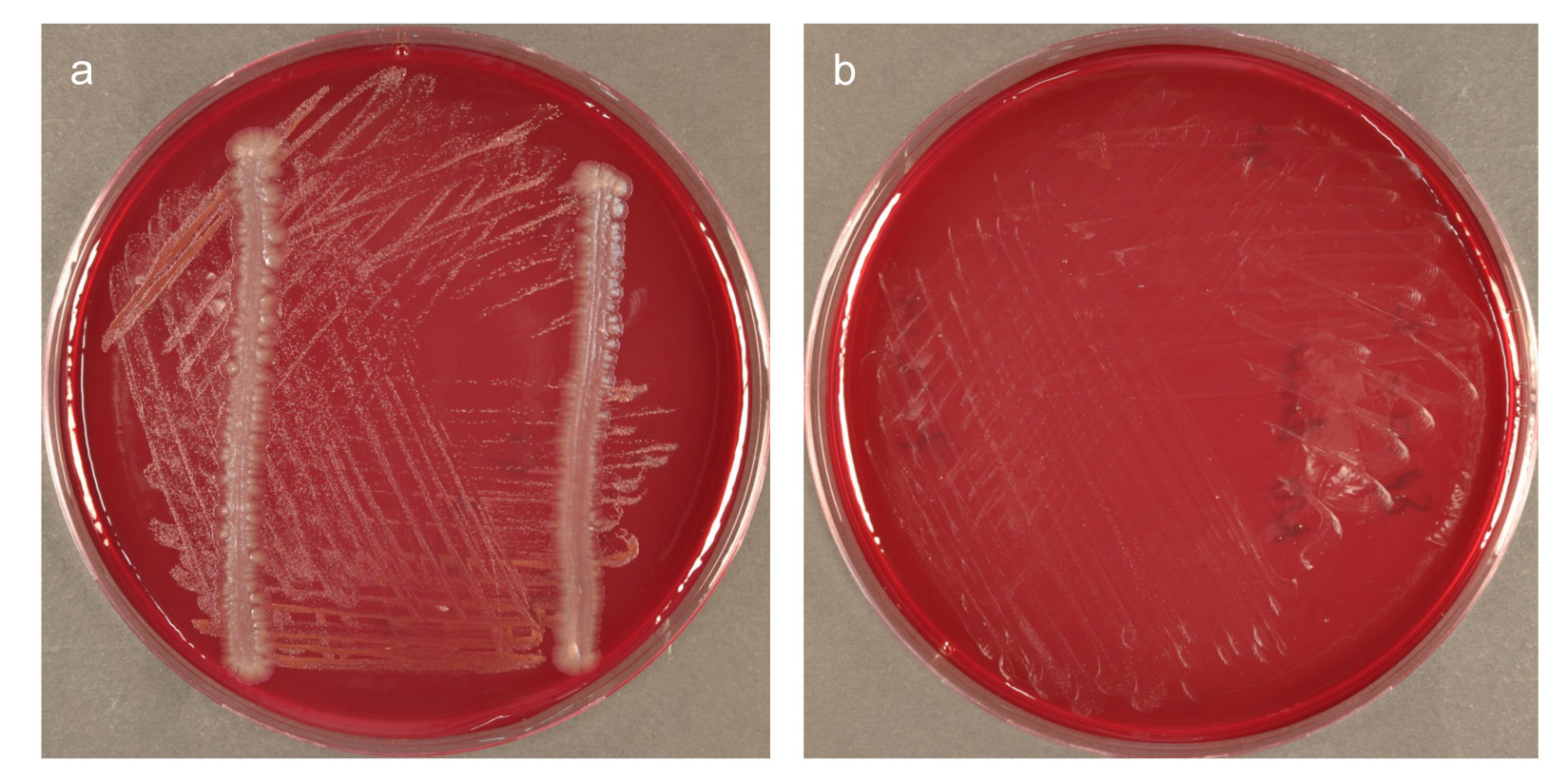
Phylum *Chloroflexi* isolate *Anaerolineae* bacterium HOT-439 is dependent on a helper strain for growth. 12-d growth: (a) with and (b) without *F*. *nucleatum* cross-streaks

Of the novel taxa that were successfully isolated in this study and survived repeated sub-culture, all were found to be dependent on helper strains for growth (Fig 3A). It was not possible to establish whether *Peptostreptococcaceae* bacterium HOT-091 strain SP19_5, which was detected after 21 days of growth as a single colony on a secondary plate but did not survive the second passage, would also have been found to be dependent on a helper strain for growth had it survived. Some of the novel taxa showed a preference for growth directly on culture media with donor cross-streaks and others showed significantly stronger growth on membranes overlying donor lawns–this suggests that the mechanism of in-vitro growth inhibition and/or stimulation of each individual difficult-to-culture strain is specific.

**Fig 4:**
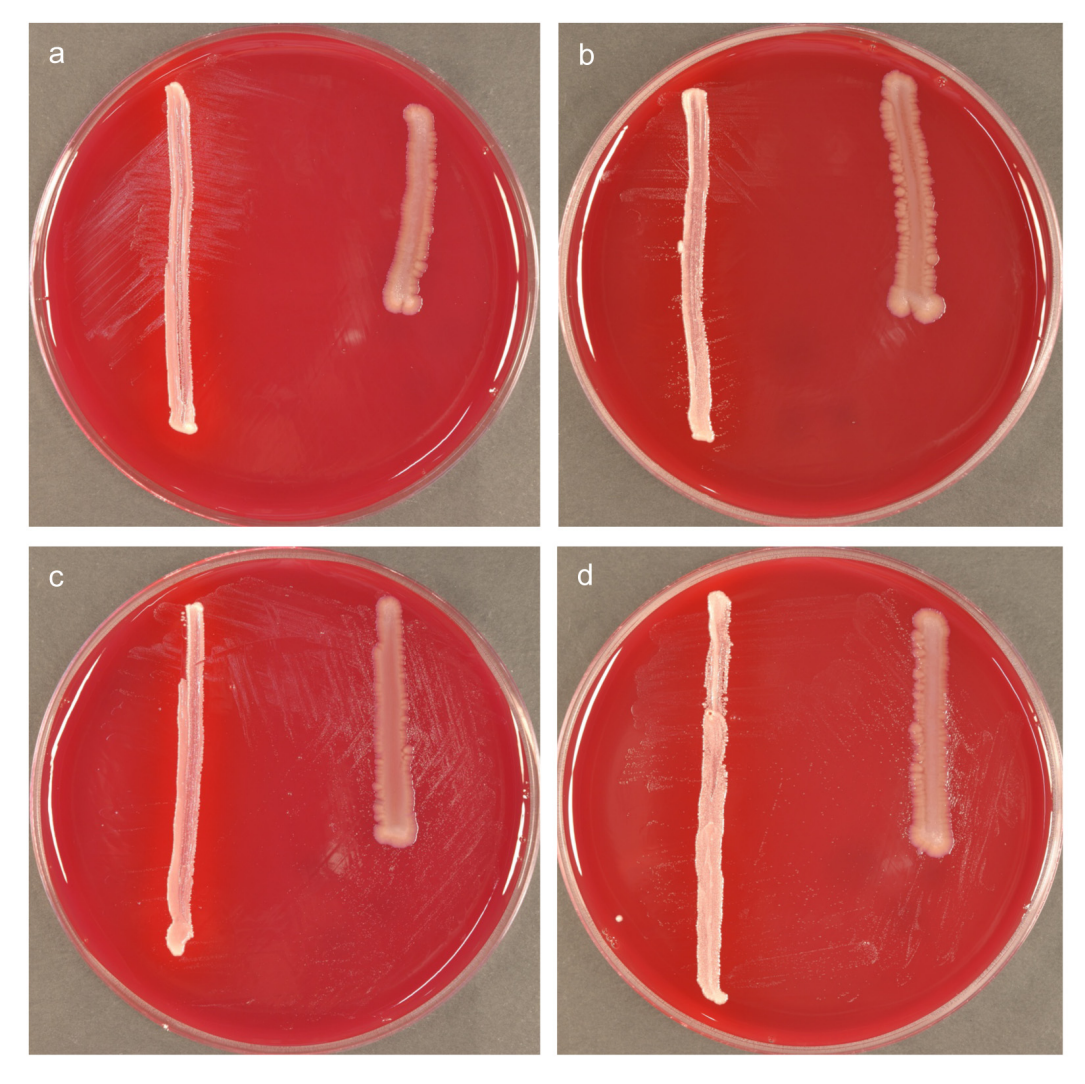
Differential growth-stimulation of *Bacteroidetes* bacterium HOT-365 in accordance with the ‘helper’ used at initial passage. Eight-day cultures with cross-streaks of *P*. *acnes* (on left) and *F*. *nucleatum* (on right). (a, c) Forth and (b, d) sixth passages after initial sub-culture on membranes over *P*. *acnes* (a-b) or *F*. *nucleatum* (c-d) lawns.

A phenomenon of growth adaptation was observed in the case of *Bacteroidetes* bacterium HOT-365, which formed a single colony beside a *P*. *acnes* streak on a secondary plate, followed by exponential growth stimulation on tertiary ‘membrane’ plates: Interestingly, where the strain had been sub-cultured on a membrane over a *P*. *acnes* lawn (at second passage), it continued to show dependence on this helper (rather than *F*. *nucleatum*) over six subsequent passages; in contrast, when the same strain had been sub-cultured first over a *F*. *nucleatum* lawn, it showed stronger growth beside cross-streaks of *F*. *nucleatum* than *P*. *acnes* over subsequent passages (Fig 4).

**Fig 5:**
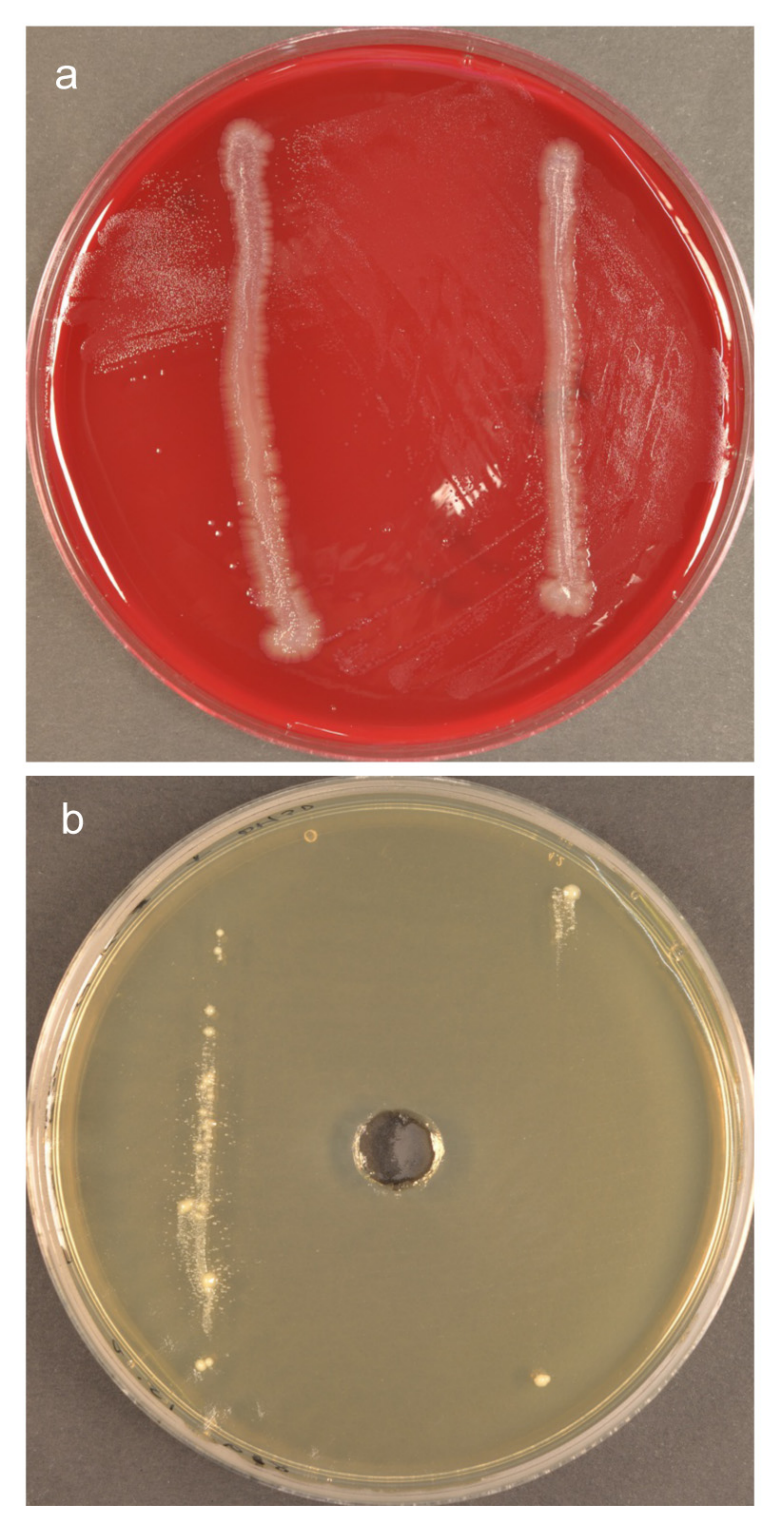
Plate cultures showing unusual helper- / dependent-strain relationships. (a) 14d-culture of *Fretibacterium fastidiosum* with bilateral cross-streaks of *F*. *nucleatum*–small circular zone of inhibition (top left) localised to an area beside the ‘helper’; (b) Five-day culture of *Prevotella* sp. HOT-376 showing self-satellitism–growth restricted to the site of, and immediately beside two vertical cross-streaks of self.

Furthermore there was variability in the effect of the ‘helper’ on growth of the ‘dependent’ strain, in some cases. Although the growth of *Anaerolineae* bacterium HOT-439 was consistently stimulated by *F*. *nucleatum*, and that of *Bacteroidaceae* bacterium HOT-272 consistently stimulated by *P*. *acnes* and *Lachnoanaerobaculum umeaense*, both *Anaerolineae* bacterium HOT-439 and *Bacteroidaceae* bacterium HOT-272 were initially stimulated by strains of *Actinomyces odontolyticus* isolated from corresponding plaque samples (Fig 2B), but inhibited by the same strains at later passages. Fig 5A shows *Fretibacterium fastidiosum*, stimulated by *F*. *nucleatum* in all areas of the streaked plate except for a small circular zone of inhibition adjacent to the top of the *F*. *nucleatum* cross streak on the left. *Prevotella sp*. HOT-376 showed evidence of self-satellitism–Fig 5B represents a spread plate of the strain, with two cross-streaks of self (rather than *F*. *nucleatum*), resulting in growth limited to the site of, and immediately beside, cross-streaks.

### Pyrosequencing analysis for the detection of previously-uncultivated/uncharacterised oral bacteria in subgingival plaque samples before and after culture

The bacterial composition of subgingival plaque samples SP9 and SP10, as well as that of 14-day cultures derived from these two samples (plaque-, pyoverdines-Fe-, and desferricoprogen-supplemented cultures, and negative control plates) were analysed by 16S rRNA gene pyrosequencing.

Both baseline plaque samples were comprised of a species-rich bacterial community (171 taxa detected amongst 8140 sequence reads from sample SP9, and 127 taxa in 6369 reads from sample SP10). Ninety-five different bacterial taxa were detected on the culture plate inoculated with a 1/100 dilution of sample SP9 sample, whilst 49–65 taxa were seen on the plates inoculated with a 1/100,000 diluted sample.

Several previously-uncultivated phylotypes were amongst the taxa detected in the plaque and culture samples (Table 2). These included phylotypes from the phyla *Actinobacteria*, *Bacteroidetes*, *Chloroflexi*, *Firmicutes*, *Fusobacteria*, *Proteobacteria*, *Spirochaetes*, *Synergistetes* and the Divisions SR1 and TM7. Altogether 58 such taxa were present, the majority were found in the baseline plaque samples and not after culture: there were 44 and 20 previously-uncultivated taxa in plaque samples SP9 and SP10 respectively, and 11 and 8 detected amongst the culture plates prepared from these samples. For example, *Fretibacterium* sp. HOT-359 and *Prevotella* sp. HOT-304 comprised 2.4–3.2% respectively of the bacterial community of plaque sample SP9, but were rarely detected in culture. There were exceptions however, and the phylotypes *Dialister* sp. HOT-119 and *Megasphaera* sp. HOT-123 were significantly more frequently detected after culture (of both plaque samples), particularly on plates supplemented with the siderophores desferricoprogen and pyoverdines-Fe; the *Megasphaera* taxon made up 10.1% of the bacterial community derived from a desferricoprogen plate culture, and 6.5–7.4% of the communities derived from pyoverdines-Fe plates, although it had comprised only 0.1–0.7% of the baseline plaque samples (Table 2). Although *Chloroflexi* taxon *Anaerolineae* bacterium HOT-439 was successfully isolated from plaque-supplemented cultures of both samples SP9 and SP10 (see section above), detection frequency by molecular methods was low (1 read (0.01%) from baseline plaque sample SP9 and none after culture–Table 2).

**Table 2:** Previously-uncultivated phylotypes detected in subgingival plaque samples and cultures derived from those samples.

	Proportion of microbiota (%)									
	Sample SP9						Sample SP10			
	Plaque sample	10^−2^ culture:Neg	10^−5^ culture:Neg	10^−5^ culture:Plaque	10^−5^ culture: Pyoverdine	10^−5^ culture: Desferricoprogen	Plaque sample	10^−2^ culture:Neg	10^−5^ culture:Plaque	10^−5^ culture: Pyoverdine
Total no. of sequences	8140	5419	9946	9519	9978	10433	6369	7730	7416	8335
*Actinomyces* HOT-414	0.01									
*Actinomyces* HOT-449	0.06									
*Actinomyces* HOT-896	0.00						0.03			
*Actinomyces* HOT-897	0.06						0.02			
*Aggregatibacter* HOT-513	0.02	0.15								
*Alloprevotella* HOT-308							0.11			
*Alloprevotella* HOT-914	0.11									
*Bacteroidetes* HOT-280	0.34	0.02								
*Bacteroidetes* HOT-365	0.11						0.05			
*Bacteroidetes* HOT-503	0.02									
*Bacteroidetes* HOT-505	0.10									
*Bacteroidetes* HOT-511	0.14									
*Bergeyella* HOT-322	0.00						0.09			
*Bergeyella* HOT-900	0.06									
*Catonella* HOT-451	0.04									
*Chloroflexi* HOT-439	0.01									
*Clostridiales* HOT-085							0.05			
*Clostridiales* HOT-093	0.54									
*Desulfobulbus* HOT-041	1.72						0.24	0.01		
*Dialister* HOT-119			0.13	0.23	1.13	1.66	0.09		0.13	1.08
*Erysipelotrichaceae* HOT-904							0.02			
*Fretibacterium* HOT-359	2.41				0.01					
*Fretibacterium* HOT-360	0.05						0.13			
*Fretibacterium* HOT-362	1.51									
*Johnsonella* HOT-166	0.12									
*Lachnospiraceae* HOT-086	0.02									
*Lachnospiraceae* HOT-500	0.14						0.05	0.10		0.01
*Leptotrichia* HOT-223	0.02	0.04								
*Megasphaera* HOT-123	0.05	0.02	0.88	1.50	6.46	10.08	0.66		1.77	7.44
*Peptostreptococcaceae* HOT-091	0.01	0.44								0.01
*Peptostreptococcaceae* HOT-103							0.02		0.28	
*Peptostreptococcaceae* HOT-369	0.01									
*Peptostreptococcaceae* HOT-383		0.81			0.04					
*Peptostreptococcaceae* HOT-495		0.09		0.01						
*Peptostreptococcaceae* HOT-790	0.02									
*Prevotella* HOT-300							0.36			
*Prevotella* HOT-301							0.02			
*Prevotella* HOT-304	3.16	0.09			0.03					
*Prevotella* HOT-309							0.06			
*Prevotella* HOT-315	0.02						0.03			
*Prevotella* HOT-443	0.86									
*Prevotella* HOT-526	1.24									
*Selenomonas* HOT-388	0.16									
*Selenomonas* HOT-501	0.01			0.01						
SR1 HOT-345	0.01									
SR1 HOT-874	0.01									
*Stomatobaculum* HOT-373	0.01									
*Stomatobaculum* HOT-910								0.01		
*Tannerella* HOT-286	0.17						0.17			
*Tannerella* HOT-808	0.05		0.03	0.01	0.01	0.01				
TM7 HOT-346							0.14			
TM7 HOT-349							0.38			
TM7 HOT-351	0.06									
TM7 HOT-356	0.01									
*Treponema* HOT-237	0.04									
*Treponema* HOT-258	0.07									
*Treponema* HOT-270	0.01									
*Veillonellaceae* HOT-132										0.01

Table 3 lists the 30 previously-cultivated, but uncharacterised taxa that were detected in baseline plaque or 14-day culture plates. They derive from the phyla *Actinobacteria*, *Bacteroidetes*, *Firmicutes* and *Spirochaetes*. In this case, the number of different taxa detected after culture (18 and 14 for samples SP9 and SP10 respectively) was comparable to that present in the baseline plaque samples (14 and 17).

**Table 3:** Previously-cultivated but uncharacterised / unnamed taxa detected in subgingival plaque samples and cultures derived from those samples.

	Proportion of microbiota (%)									
	Sample SP9						Sample SP10			
	Plaque sample	10^−2^ culture: Neg	10^−5^ culture: Neg	10^−5^ culture: Plaque	10^−5^ culture: Pyoverdine	10^−5^ culture: Desferricoprogen	Plaque sample	10^−2^ culture: Neg	10^−5^ culture: Plaque	10^−5^ culture: Pyoverdine
Total no. of sequences	8140	5419	9946	9519	9978	10433	6369	7730	7416	8335
*Actinobaculum* HOT-183							0.19			
*Actinomyces* HOT-169							0.55	0.01	0.07	
*Actinomyces* HOT-171	0.01	0.06				0.01	0.13		0.05	0.06
*Actinomyces* HOT-172		0.02								
*Actinomyces* HOT-175							0.05			
*Actinomyces* HOT-178			0.02				0.03		0.03	0.08
*Actinomyces* HOT-180	0.05	0.42	0.02	0.46	0.18	0.29	1.15	0.48	0.15	1.16
*Actinomyces* HOT-448							0.19			
*Bacteroidaceae* HOT-272	0.32	0.02		0.07	0.01	0.54	0.08		2.51	0.82
*Bacteroidales* HOT-274	0.06						0.79	0.01	4.38	
*Capnocytophaga* HOT-323					0.04				0.03	0.01
*Capnocytophaga* HOT-324		0.06								
*Capnocytophaga* HOT-326	0.41		0.03							
*Capnocytophaga* HOT-336	0.05		0.03	0.02	0.01	0.24	0.03		0.03	0.02
*Capnocytophaga* HOT-864		0.04	0.02	0.02	0.01	0.12	0.08			
*Peptococcus* HOT-167	0.04									
*Peptoniphilus* HOT-386		0.02			0.01					
*Peptostreptococcaceae* HOT-081	0.15	0.02	0.01							
*Peptostreptococcaceae* HOT-106								0.09		
*Peptostreptococcaceae* HOT-113	0.31	0.74	0.03	0.01	0.02	0.11				
*Prevotella* HOT-306							0.06			
*Prevotella* HOT-317	0.33				0.01		0.09			0.02
*Prevotella* HOT-820		0.02								
*Streptococcus* HOT-058		0.06		0.03		0.01				
*Streptococcus* HOT-064							7.99	0.13		0.36
*Streptococcus* HOT-070							0.20	0.01		
*Streptococcus* HOT-071	1.04	4.69	31.80	10.70	1.56	11.85				
*Treponema* HOT-268	0.06									
*Veillonellaceae* HOT-129	0.04	0.04					0.03			
*Veillonellaceae* HOT-155	0.05						0.02			0.01

Some sequences were returned ‘unclassified’ by the mothur wang method, either because they did not match sequences in the HOMD database or because they could not be unambiguously identified. These were re-analysed by BLAST comparison with the HOMD extended set and GenBank nucleotide databases. This led to the identification of a further 23 previously-uncultivated phylotypes (listed in Table 4), 13 of which were detectable after culture. There were an additional three phylotypes with less than 98.5% 16S rRNA gene sequence similarity to any human oral taxon in the HOMD extended set, but which matched full-length 16S rRNA gene sequences available in the GenBank nucleotide database. These taxa have been assigned HOMD designations as follows: *Aggregatibacter* sp. HOT-949 (Genbank accession no. JF506655); five sequences representing a novel taxon within the family *Peptostreptococcaceae* (AM419965), assigned the designation *Peptostreptococcaceae* bacterium HOT-950; and *Treponema* sp. HOT-951 (AM420103).

**Table 4:** ‘Unclassified’ sequences representing previously-uncultivated phylotypes detected in subgingival plaque samples and cultures derived from those samples.

	Proportion of microbiota (%)									
	Sample SP9						Sample SP10			
	Plaque sample	10^−2^ culture: Neg	10^−5^ culture: Neg	10^−5^ culture: Plaque	10^−5^ culture: Pyoverdine	10^−5^ culture: Desferricoprogen	Plaque sample	10^−2^ culture: Neg	10^−5^ culture: Plaque	10^-5^culture: Pyoverdine
Total no. of sequences	8140	5419	9946	9519	9978	10433	6369	7730	7416	8335
*Actinomyces* HOT-B78	0.04	0.02	0.01				0.03	0.01		
*Aggregatibacter* HOT-E49	0.02	0.07								
*Centipeda* HOT-B01	0.06									
*Centipeda* HOT-D18	0.06									
*Clostridiales* HOT-G74	0.01									
*Fretibacterium* HOT-360	0.12						0.06			
*Fretibacterium* HOT-362	0.25									
*Fretibacterium* HOT-453							0.05			
*Fusobacterium* HOT-A71	0.76	1.94		0.03	0.01					
*Fusobacterium* HOT-H27							0.17			0.02
*Herbaspirillum* HOT-A32		0.04						0.01		
*Lachnospiraceae* HOT-F93	0.02	0.31								
*Lachnospiraceae* HOT-G33	0.04									
*Leptotrichia* HOT-B57	0.01									
*Selenomonas* HOT-E44	0.01	0.35					0.02			
*Selenomonas* HOT-F19	0.04							0.01		
*Selenomonas* HOT-F21	0.06	0.06								
*Selenomonas* HOT-G00	0.01									
*Selenomonas* HOT-G51		0.04					0.02			
*Selenomonas* HOT-G67							0.11		0.39	
*Streptococcus* HOT-C65	0.01	0.07			0.88		0.16	1.20		0.10
*Streptococcus* HOT-E30										0.01
*Veillonellaceae* HOT-135	0.01									

## Discussion

A targeted approach (using rRNA-directed colony hybridisation) for the cultivation of specific novel ‘uncultivated’ oral taxa, has been used successfully in the past, leading to isolation of the taxa *Lachnospiraceae* sp. HOT-500 and *Fretibacterium fastidiosum* [19, 21]. In an attempt to develop an *in-vitro* culture model for the isolation, in parallel, of more than one novel taxon, an open-ended approach was used in this study. On the basis that truly difficult-to-culture organisms may depend for growth on signal interaction and metabolic cooperation with other bacteria within the biofilm community that they inhabit, undiluted suspensions of plaque were placed in central wells in agar culture plates, to stimulate growth of previously-uncultivated oral bacteria in heavily-diluted subgingival plaque samples, inoculated onto the periphery of the plates. In addition, siderophores were used as potential growth supplements. Colonies of interest on mixed primary plates were empirically selected according to size (microcolonies) or position (appearance of satellitism). Whilst none were derived from negative control plates, 77 such colonies were identified on plaque-, pyoverdines-Fe-complex- and desferricoprogen-supplemented plates, and several of these were later found to represent novel taxa or difficult-to-culture strains. Ultimately, this culture model led to the successful isolation of five previously-uncultivated strains, representatives of three novel taxa, including *Anaerolineae* bacterium HOT-439, the first oral taxon from the *Chloroflexi* phylum to have been cultivated.

In line with our expectation that previously-uncultivated taxa would be dependent on a biofilm or community lifestyle, the novel strains were found to require ‘helper’ bacteria for growth *in vitro*. The *Anaerolineae* bacterium HOT-439, *Bacteroidetes* bacterium HOT-365 and *Bacteroidaceae* bacterium HOT-272 isolates were found to be dependent on one or both of the helper bacteria *P*. *acnes* and *F*. *nucleatum*, as well as, in the case of *Bacteroidaceae* bacterium HOT-272, *Lachnoanaerobaculum umeaense*, which was found locally in the mixed community. Traditionally the relationship between helper and recipient strain is a most specific one, as a result of a dependence on the helper for the provision of specific chemical factors [16, 17] or a change in the environment [22]. He *et al* [7] have demonstrated host specificity, even to the level of strain, for the parasitic lifestyle of the recently-cultivated taxon TM7x. *A*. *odontolyticus* strain XH001, but not *A*. *odontolyticus* ATCC 17982 was suitable as a host. Most interesting however, was the finding in our study that, certain ‘helpers’, namely two *A*. *odontolyticus* strains isolated from plaque of different subjects, appeared to have a variable effect on *Anaerolineae* bacterium HOT-439 and *Bacteroidaceae* bacterium HOT-272 (initially stimulating and later consistently inhibiting growth). To our knowledge, such a phenomenon has not been described before, and the implication here, is of a change in the phenotype of *A*. *odontolyticus* on repeated culture, rather than a change in that of the recipient strains, which were consistently stimulated by the other helpers.

*Bacteroidetes* bacterium HOT-365 became ‘domesticated’, growth becoming stronger on repeated sub-culture, particularly when cultured on membranes over helper lawns, indicating that growth stimulatory molecules small enough to pass through 0.45 μm pores are involved. Furthermore, this single recipient strain showed a remarkable adaptive capacity, with ability to derive growth-stimulatory factors from different helpers based on the ‘helper’ used at an earlier passage. Similar examples of bacterial phenotypic plasticity, with the development of growth characteristics following adaptation to growth conditions, have been reported previously [13, 23, 24].

Self-satellitism was observed in the case of *Prevotella* sp. HOT-376. This phenomenon may be explained by the requirement for a critical mass of cells to be present for successful colony formation. It is well known that for the growth of many bacterial species in broth culture a threshold number of cells are required, necessitating the use of a starter culture. The importance of cell density for growth has also been demonstrated for the dependent strain *Prochlorococcus* MIT9215 [25].

This study confirmed, in a controlled experiment, that certain siderophores have a stimulatory role in the growth of the difficult-to-culture strains *F*. *fastidiosum* and *Prevotella* sp. HOT-376. Additional evidence for the growth-enhancing effect of pyoverdines-Fe-complex and desferricoprogen, specifically for the as-yet-uncultivated phylotypes *Dialister* sp. HOT-119 and *Megasphaera* sp. HOT-123, was provided by analysis of pyrosequencing data from subgingival plaque samples, which indicated a considerably higher prevalence of these taxa in the 14-day bacterial communities of siderophore-supplemented cultures, than in the baseline plaque samples or cultures not supplemented with siderophores. The implication is that the ability to synthesise these factors has been lost in certain ‘uncultivable’ bacteria, a concept suggested also by Lewis *et al* [13]. D’Onofrio *et al* [16] showed that the addition of siderophores to media resulted in growth promotion of previously uncultured bacteria from marine sediment, with desferricoprogen showing the broadest range of growth induction of the siderophores tested. Likewise, Guan and Kamino [26] found that addition of the siderophore desferrioxamine to culture media led to the formation of a more species-rich bacterial community, and an increased yield of novel isolates from the marine environment. Although the primary function of siderophores is to scavenge ferric (III) ions for the solubilisation and uptake of iron, a process most relevant in aerobic environments, the finding in this study that siderophores also act as growth factors for anaerobic bacteria, implies a diverse effect unrelated to iron availability and acquisition under these conditions. One possibility is that siderophores have an important role in electron transport processes that facilitates growth of anaerobic bacteria. Furthermore the production of pyoverdine has been linked to biofilm formation [27–30], suggesting a possible role in growth promotion. Further investigation into the potential mechanisms involved in the growth-enhancing effects of siderophores and helper strains is warranted.

Five novel bacterial strains, representatives of three previously-uncultivated taxa, were isolated in this study (although *Peptostreptococcaceae* bacterium HOT-091 survived only one passage). As a consequence of having generated pure cultures of these novel bacteria, there is now opportunity to comprehensively characterise the phenotype and genotype of the strains, as well as to assess them for association with disease/virulence potential.

Of the novel taxa isolated, *Anaerolineae* bacterium HOT-439 is of particular interest in that it represents the first member of the phylum *Chloroflexi* from the human oral cavity to have been cultivated. Each of the three strains was isolated from a different subject. Based on molecular analyses, it has been suggested that the *Chloroflexi* taxon is a low-abundance member of the human oral cavity [5, 31], with a prevalence of 0.003% (HOMD). Likewise, in our study, this taxon was infrequently detected by pyrosequencing. However, due to the presence of base mismatches between the 16S rRNA gene sequence of *Anaerolineae* bacterium HOT-439 and two commonly used ‘universal’ bacterial primers, 1525R and 1492R (the latter of which was used in this study), these findings may be underestimates and the result of a molecular detection bias against this taxon [6]. Indeed, the finding that this strain was successfully isolated from three of the four subgingival plaque samples harvested from deep periodontal pockets indicates that the oral *Chloroflexi* taxon may not be as rare as originally thought, at least in this ‘disease-associated’ habitat. Abusleme *et al* [32] reported a markedly higher prevalence of *Chloroflexi* taxon HOT-439 in subgingival sites of patients with chronic periodontitis, than of periodontally-healthy subjects, and assigned this taxon as a member of the ‘core’ periodontitis-associated microbiome. Furthermore, Szafranski et al [33] found, using the Area Under the Curve (AUC) method, that *Chloroflexi* taxon HOT-439 was the only potential biomarker for periodontitis showing statistical significance. To date, only single-cell-derived, partial genomes are available for *Chloroflexi* taxon HOT-439 and it will be useful to sequence the complete genome from the pure cultures isolated here. Inferences from the available genomic data imply a niche-specific adaptation for survival in host environments, with regard to host evasion and the ability to scavenge material from lysed cells/tissue [31], which corresponds well with the implication that this taxon plays a role in periodontitis.

It is interesting to note that other taxa assigned by Abusleme *et al* [32] to the ‘core’ periodontitis-associated microbiome included, not only the potential keystone pathogen *Porphyromonas gingivalis* [34], but also several difficult-to-culture/as-yet-uncultivated taxa, such as *Peptostreptococcaceae* bacterium HOT-091, *Fretibacterium fastidiosum* and other *Fretibacterium* species, and TM7 phylotypes. Despite being potentially fastidious and resistant to in-vitro culture, as-yet-uncultivated taxa may nevertheless play important roles in disease. Hence the quest to cultivate and study such novel bacteria remains a high priority for the development of our understanding of the human oral microbiome in health and disease.

## Material and Methods

### Growth stimulation of ‘difficult-to-culture’ oral bacterial strains by siderophores

Lawn cultures of *Prevotella* sp. HOT-376 (KCL-E7_34) were prepared by inoculating plates of Fastidious Anaerobe Agar (FAA, Lab M, UK) with 50 μl suspensions (McFarland standard 1 turbidity), prepared from a four-day plate culture. Plates of blood Agar Base No. 2 (Lab M, UK) + 5% horse blood (BA) were inoculated with 50 μl suspensions (McFarland standard 4) of *Fretibacterium fastidiosum* DSM 25557^T^ from a seven-day plate culture. Test agents were placed in a central well in each plate in 1.5, 15 or 150 μg amounts. The agents tested included the siderophores desferricoprogen (EMC Microcollections, Germany), pyoverdines-Fe-complex (Sigma-Aldrich, UK) and ferrichrome-Fe-free (Santa Cruz, USA); siderophore-like molecules 2,3-dihydroxybenzoic acid (Acros Organics, Belgium) and salicylic acid (ChemCruz Biochemicals, USA); and the iron chelator ferric citrate (Sigma-Aldrich, UK). The positive control was filtered supernatant of a 72-hr broth culture of *F*. *nucleatum* and negative controls were the agent diluents. Plates were incubated in an anaerobic workstation (Don Whitley Scientific Ltd.) with an atmosphere of 80% nitrogen, 10% hydrogen and 10% carbon dioxide at 37°C for up to 35 days. The diameter and relative density of any zone of growth were recorded, and used to arbitrarily grade growth stimulation relative to positive (++++) and negative controls (-). The experiment was performed in triplicate.

### Development of an in-vitro culture model for the cultivation and isolation of previously-uncultivated oral bacteria

#### Subjects and samples

Four subjects with chronic periodontitis, none of whom had received periodontal or antimicrobial therapy within the previous three months, were recruited for the study with their informed written consent. Ethical approval was granted by the South West London REC 3 Research Ethics Committee (REF: 10/H0803/161). Subgingival plaque samples were collected with a sterile curette from two deep (7–9 mm) periodontal pockets from each subject, pooled and suspended in Reduced Transport Medium (RTM) [35].

#### Culture of plaque samples

Samples were transported to an anaerobic workstation within 1 hr of collection. After vortexing for 1 min, a 10-fold dilution series of each sample was prepared in RTM. Pre-reduced BA plates were inoculated with 50 μl of each 10^2^-, 10^5^- and 10^6^-diluted plaque suspension. 150 μl neat plaque suspension (matched to the plaque that was diluted and used for culture), 15 μg siderophore (pyoverdines-Fe or desferricoprogen), or 150 μl sterile water (negative control) were added to a central well in the plates, and plates were incubated anaerobically.

#### Sub-culture and identification of colonies of interest in mixed cultures

After 8–28 days of incubation, culture plates were inspected under a dissecting microscope and any colonies of interest (namely microcolonies or colonies showing satellitism: growing either around or on larger colonies) were passaged onto: (a) fresh BA plates, cross-streaked with helper strains (*Propionibacterium acnes* ATCC 6919, *Fusobacterium nucleatum subspecies polymorphum* NCTC 10562, or colonies in proximity to the colonies of interest) and where relevant, supplemented with either pyoverdines-Fe or desferricoprogen; or (b) cellulose acetate membranes overlying lawn cultures of the helper strains. Once pure, sub-cultured colonies were identified by partial 16S rRNA gene sequencing with primer 519R (as described previously [36]) after PCR of extracted DNA or ‘touch’-PCR of single colonies with ‘universal’ primers 27FYM and 1492R [37]. For any taxa: (a) found to have less than 98.5% similarity to 16S rRNA gene sequences in the Human Oral Microbiome (HOMD) and GenBank nucleotide databases, or (b) with more than 98.5% 16S rRNA gene sequence similarity to previously-uncultivated phylotypes, clone libraries were prepared (as described previously [36]) from amplicons representing 16S rRNA gene ‘touch’-PCR of single colonies with ‘universal’ primers; and 20 clone inserts were sequenced per library to confirm purity. The full length of the 16S rRNA gene for any such novel taxon was sequenced with multiple primers for triple coverage [36].

#### Pyrosequencing analysis of subgingival plaque samples before and after culture

500 μl of neat plaque suspension from subjects SP9 and SP10 were subjected to propidium monoazide (PMA) treatment prior to DNA extraction. PMA dye is cell membrane-impermeable and selectively binds to/modifies DNA from cells with damaged cell membranes; consequently, only DNA from intact cells was amenable to PCR amplification later [38]. Briefly, 1.25 μl of 20 mmol/L PMA (Cambridge Biosciences, UK) was added to the plaque suspensions, which were incubated in the dark with occasional agitation (5 min) and then placed on ice under a 500 W halogen lamp for 3 min, with occasional agitation. DNA was extracted from the samples using the GenElute Bacterial Genomic DNA kit (Sigma-Aldrich, UK), following the protocol for Gram positive bacteria.

At 14 d of growth, colonies were harvested from half of the surface of culture plates derived from 10^2^- and 10^5^-dilutions of samples SP9 and SP10, and suspended in 2 ml PBS. 500 μl of each suspension was PMA-treated and DNA was extracted as described above.

An approximately 500 bp region of the 16S rRNA gene (covering V1–V3) was PCR-amplified from extracted DNA samples using composite fusion primers comprising universal 16 S primers (27FYM and 519R) along with Roche GS-FLX Titanium Series adapter sequences (A & B) for 454 pyrosequencing using the Lib-L emPCR method. Previously described unique 12 base error-correcting Golay barcode sequences [39] were incorporated into the forward primers (5′-CCATCTCATCCCTGCGTGTCTCCGACTCAG-NNNNN NNNNNNN-AGAGTTTGATYMTGGCTCAG-3′). The appropriate barcoded A-27FYM and the B-519R (5′-CCTATCCCCTGTGTGCCTTGGCAGTCTCAG-GWATT ACCGCGGCKGCTG-3′) primers were used in PCRs with Extensor Hi-Fidelity PCR Mastermix (Thermo Scientific). There was an initial denaturation step of 5 mins at 95°C followed by 25 cycles of 95°C for 45 s, 53°C for 45 s, 72°C for 1 m 30 s and a final extension of 72°C for 15 mins. PCR amplicons were subsequently purified using the QIAquick PCR purification kit (Qiagen) following the manufacturer's instructions. The size and purity of the amplicons was checked using the Agilent DNA 1000 kit and the Agilent 2100 Bioanalyzer. The amplicons were quantitated by means of a fluorometric assay using the Quant-iT Picogreen fluorescent nucleic acid stain (Invitrogen) and then pooled at equimolar concentrations (1×10^9^ molecules/μl). emPCR and undirectional sequencing of the libraries was performed using the Lib-L kit and Roche 454 GS-FLX Plus Titanium chemistry by the the Department of Biochemistry, University of Cambridge, UK. The mothur pipeline [40] was used for denoising, trimming, alignment to the Silva reference alignment and removal of chimerae. Sequences were identified using the classify.seqs command.

#### Accession numbers

Partial 16S rRNA gene sequences for the novel taxa detected in this study have been submitted to the DDBJ/EMBL/GenBank databases under the following accession numbers: *Bacteroidetes* bacterium HOT-365 SP18_29, KT861603; *Chloroflexi* taxon *Anaerolineae* bacterium HOT-439 SP9_5, KT861598; *Chloroflexi* taxon *Anaerolineae* bacterium HOT-439 SP10_2, KT861597; *Chloroflexi* taxon *Anaerolineae* bacterium HOT-439 SP19_9, KT861599.
